# Comparative and developmental anatomy of the fibularis brevis muscle: morphological variants and their clinical significance

**DOI:** 10.3389/fcell.2025.1647407

**Published:** 2025-08-14

**Authors:** Łukasz Olewnik, Ingrid C. Landfald, Daria Domosławska, Kacper Ruzik, George Triantafyllou, Maria Piagkou, Teresa Vázquez

**Affiliations:** ^1^ Department of Clinical Anatomy, Mazovia Academy in Płock, Płock, Poland; ^2^ “VARIANTIS” Research Laboratory, Department of Clinical Anatomy, Mazovian Academy in Płock, Płock, Poland; ^3^ Department of Anatomy, Faculty of Medicine, National and Kapodistrian University of Athens, Athens, Greece; ^4^ Donor Body Center, Complutense University of Madrid, Madrid, Spain

**Keywords:** fibularis brevis, anatomical variation, tendon insertion, embryology, fibularis digiti quinti, musculoskeletal imaging, comparative anatomy, surgical anatomy

## Abstract

**Background:**

The fibularis brevis muscle (FBM)is a key stabilizer of the lateral ankle, yet its anatomy exhibits a notable degree of variability. While often overshadowed by the fibularis longus, FBM and its tendon (FBT) play critical roles in foot eversion, proprioception, and surgical reconstruction. However, inconsistent terminology and limited integrative studies have hindered comprehensive clinical understanding.

**Methods:**

This review synthesizes data from developmental anatomy, fetal and adult cadaveric dissections, comparative morphology across vertebrates, and clinical imaging. Anatomical classifications of the FBT and fibularis digiti quinti (FDQ) were evaluated alongside their embryological origins, phylogenetic trends, imaging correlates, and surgical relevance.

**Results:**

A unified classification of FBT (Types I–IV) and FDQ (Types 1–3) is proposed, reflecting morphological, developmental, and radiological patterns. The FBM muscle demonstrates modular variability that parallels phylogenetic adaptations from complete absence in certain cursorial mammals to hypertrophy in arboreal primates. Variant tendinous insertions and accessory fascicles may mimic pathology in MRI or complicate surgical dissection.

**Conclusion:**

FB represents a morpho-evolutionary continuum rather than a static anatomical unit. Recognition of its variants through improved classification, imaging protocols, and evolutionary insight is essential for anatomists, radiologists, and surgeons. This integrative approach advances the clinical and biological understanding of lateral leg musculature.

## 1 Introduction

### 1.1 Overview of the fibularis brevis muscle

The fibularis brevis muscle (FBM), also referred to as peroneus brevis, is one of the two principal muscles located in the lateral compartment of the leg. It originates from the distal two-thirds of the lateral surface of the fibula and inserts onto the tuberosity of the fifth metatarsal (Bergman et al., 1988; Moore et al., 2017). It is innervated by the superficial fibular nerve and plays a crucial role in eversion of the foot and in maintaining balance during locomotion, especially on uneven surfaces (Otis et al., 2004; Verma et al., 2011).

### 1.2 Relevance in lateral ankle stability, tendon transfer, and chronic instability

Clinically, the FBM and its tendon (FBT) are essential stabilizers of the lateral ankle joint. They contribute significantly to dynamic ankle control and are frequently involved in overuse injuries, lateral ankle sprains, and chronic ankle instability (Alanen et al., 2001; Philbin et al., 2009). FBT pathologies, including tears, subluxation, and tenosynovitis, are common in athletes and runners (Dombek et al., 2003; Seybold et al., 2016). Moreover, the FBT is a preferred graft source in various reconstructive procedures involving the lateral ankle complex, especially in cases of irreparable fibulariss longus or FBT ruptures (Clement et al., 2019; Housley et al., 2017; Olewnik et al., 2020).

Recent reports have also highlighted the surgical utility of accessory FBT slips and related variants, such as fibularis digiti quinti (FDQ) and fibularis quartus, as autografts in tendon reconstruction (Carlis et al., 2017; Yammine, 2015). However, anatomical variability of the FBT and its derivatives remains underreported in standard clinical guidelines and often underappreciated in surgical planning.

### 1.3 Diagnostic pitfalls in imaging; overlap with fibularis longus

Radiological evaluation of FBM pathology poses diagnostic challenges, as accessory tendinous structures and variant insertion patterns can mimic longitudinal tears or soft-tissue masses (Sookur et al., 2008; Vohra et al., 2011). The FBM is frequently mistaken for fibularis longus in both static MRI and dynamic United States, especially in the presence of bifid tendons or accessory fascicles (Olewnik et al., 2020; Ruzik et al., 2022). Moreover, FDQ and fibularis quartus may be misinterpreted as abnormal masses if not properly recognized in radiological imaging (Chaney et al., 2017; Demir et al., 2015).

### 1.4 Aim

The aim of this review is to provide a comprehensive and structured reevaluation of the FBM, integrating data from developmental anatomy, evolutionary morphology, anatomical variation, comparative interspecies analysis, and clinical radiological findings. By synthesizing cadaveric, fetal, and imaging-based studies, we propose a unified framework for understanding the morphological variants of FB and their implications in clinical and surgical contexts (Diogo et al., 2010a; Olewnik et al., 2023; Olewnik et al., 2020).

## 2 Developmental and evolutionary origins

### 2.1 Embryogenesis: FB arises from the dorsal muscle mass

The FBM develops from the dorsal muscle mass of the lower limb bud, which is responsible for forming the extensors and evertors of the lower extremity (Bardeen, 1906; Bardeen and Lewis, 1901). By the fifth week of gestation, myogenic precursor cells from the somites migrate into the developing limb bud, where they begin to organize into distinct compartments. FB originates posterolaterally and forms in parallel with fibularis longus, although each follows an independent developmental trajectory (Moore et al., 2017; Moore et al., 2020).

During fetal development, FBM gradually separates from fibularis longus and fibularis tertius, assuming its mature anatomical position and forming the myotendinous junction that inserts into the base of the fifth metatarsal. This differentiation occurs concomitantly with the medial rotation of the limb and is visible in second-trimester dissections (Olewnik et al., 2023).

### 2.2 Regulatory molecules: Pax3, Myf5, MyoD

The ontogeny of FBM, like other limb muscles, is governed by a well-characterized hierarchy of myogenic regulatory factors (MRFs). Pax3 initiates migration of progenitor cells from the dermomyotome, while Myf5 and MyoD drive their commitment and differentiation into skeletal muscle (Diogo et al., 2010a). These transcription factors are activated in response to limb-level morphogen gradients including FGF, Wnt, and BMP.

Although FBM specific expression studies remain limited, analysis of dorsal vs. ventral muscle development suggests that the posterolateral domain of the limb bud expresses a distinct profile of MRFs, facilitating formation of fibular musculature (Albay and Candan, 2017). Alterations in this tightly controlled sequence may lead to aberrant muscular differentiation, malformations, or asymmetries.

### 2.3 Errors in segmental patterning and migration as sources of anatomical variants

Disruptions in the developmental program can result in morphological anomalies of FBM. Errors in somite segmentation, progenitor migration, or fascial compartmentalization may lead to supernumerary muscle slips, atypical trajectories, or duplications. Embryologically, these anomalies may result from incomplete apoptosis of primitive muscle condensations or persistence of transient myogenic bridges.

Variants of FBM such as additional tendinous slips, aberrant insertions, or fusion with adjacent muscles are frequently reported in both adult and fetal specimens (Bhargava et al., 1961; Musiał, 1963; Olewnik et al., 2023). These findings support a developmental origin for many observed variations and highlight the need for embryological awareness in interpreting FBM anatomy. Although the anatomical variants of the FBT such as bifurcated (Type II) insertions are well-documented developmentally, no current evidence links these patterns to specific genetic mutations or congenital syndromes in humans. They are presently regarded as sporadic developmental anomalies rather than heritable traits. Nonetheless, further investigation into potential genetic predispositions may be warranted. Future molecular studies could clarify whether certain transcriptional or epigenetic variations subtly influence the formation or persistence of these morphotypes (Olewnik et al., 2023; Olewnik et al., 2020).

### 2.4 Evolutionary persistence of muscle modularity

The development of FBM exemplifies the concept of muscle modularity, whereby muscles can evolve and adapt as discrete anatomical units. This modularity allows for plasticity in form and function across taxa, as muscles can undergo hypertrophy, fusion, or even regression without disrupting the global musculoskeletal architecture (Abdala and Diogo, 2010; Diogo et al., 2010a).

In the case of FBM, such modularity explains the presence of accessory fibular muscles in some individuals and their complete absence in others. These variations do not necessarily impair function but reflect an evolutionarily conserved mechanism for localized adaptation to biomechanical demands (Poonia et al., 2020; Yammine, 2015).

### 2.5 Phylogenetic background: FB as remnant of lateral digital extensors

From a phylogenetic perspective, FBM likely originated from lateral digital extensor muscles that were prominent in early tetrapods. In non-human primates, especially arboreal species, FBM is robust and contributes significantly to dynamic foot stabilization and prehensile support. In contrast, terrestrial quadrupeds such as carnivores or ungulates often exhibit a reduced or absent FB, having functionally redistributed eversion roles to other muscles (Diogo et al., 2010a).

This cross-species variability highlights FBM as a developmentally plastic and evolutionarily responsive muscle, shaped by postural requirements and gait mechanics. In modern humans, its persistence in a distinct fascial plane with separate innervation reflects both its ancestral roots and its importance in maintaining lateral ankle integrity.

## 3 Typical morphology of the fibularis brevis

### 3.1 Origin, insertion, fascial and neurovascular context

The FBM is a key component of the lateral compartment of the leg. It originates from the distal two-thirds of the lateral surface of the fibula, as well as from the anterior intermuscular septum and deep fascia. The muscle fibers converge into a strong tendon that runs distally and posterior to the lateral malleolus, finally inserting onto the tuberosity at the base of the fifth metatarsal (Moore et al., 2017; Zielinska et al., 2024). The FBM is enveloped in a common fascial sheath with the fibularis longus, with which it shares the lateral fibular groove and peroneal tendon sheath (Olewnik et al., 2023; Olewnikvet al., 2020).

• The muscle is innervated by the superficial fibular nerve, a branch of the common fibular nerve, and is vascularized by perforating branches of the fibular artery, (van Dijk et al., 2016). The presence of a myotendinous junction typically occurs in the lower third of the leg (Mateen et al., 2022).
• a detail that is clinically relevant in surgical planning and tendon harvesting procedures.

### 3.2 Relationship to peroneal retinacula, sural nerve, lateral malleolus

Anatomically, the FBT is stabilized at the lateral ankle by the superior and inferior peroneal retinacula. These fibrous bands prevent subluxation or dislocation of the tendon during foot eversion. The superior peroneal retinaculum extends from the posterior border of the lateral malleolus to the calcaneus, forming the primary retaining structure for FBT and fibularis longus tendons. The inferior retinaculum, more complex in configuration, contributes to the pulley-like function of the retromalleolar path (Sookur et al., 2008).

Laterally, the FBT is in close proximity to the sural nerve and its lateral dorsal cutaneous branch, as well as to the small saphenous vein. The proximity of these structures poses a risk during lateral surgical approaches and must be considered during peroneal tendon repairs or reconstructions (Philbin et al., 2009). The peroneal tubercle on the lateral calcaneus serves as a distinguishing anatomical landmark separating the tendons of FBM and fibularis longus distally (Macalister, 1875).

### 3.3 Normal imaging anatomy on MRI and ultrasound

On MRI, the FBT is visualized as a well-defined, low-signal-intensity structure on T1- and T2-weighted images. It courses posterior to the lateral malleolus and can be identified separately from the FLT in axial and coronal planes. Its normal myotendinous junction appears as a smooth, fusiform transition zone with intermediate signal, located proximally to mid-calf (Vohra et al., 2011).

In ultrasonography, FBT appears as a homogeneous echogenic fibrillar structure within the shared fibularis tendon sheath. Dynamic scanning can confirm the integrity of its gliding movement and detect intrasheath subluxation or early tendinopathic changes. Proper visualization requires axial and longitudinal planes just posterior to the fibula and around the retromalleolar region (Carlis et al., 2017; Dombek et al., 2003).

Recognition of the FBT’s normal anatomy on imaging is crucial to avoid misdiagnosing bifid tendons, longitudinal splits, or accessory insertions as pathological findings (Seybold et al., 2016; Yammine, 2015).

## 4 Anatomical variants and classification systems

### 4.1 Fibularis brevis tendon types

In a comprehensive cadaveric study of 102 lower limbs, the FBT was found to be present in all specimens (Olewnik et al., 2020). Two main types of distal insertion were identified based on the number and morphology of terminal tendon slips: a single insertion (Type I) and a bifurcated pattern (Type II). Type II was further subdivided into three morphologically distinct subtypes based on the course and insertion of the accessory tendinous band.

#### 4.1.1 Type I–single distal attachment

This configuration consisted of a single tendon inserting into the tuberosity at the base of the fifth metatarsal bone on its lateral surface. It was the most commonly observed variant, present in 72 cases (70.6%). This pattern is consistent with the classic textbook description and is considered the standard anatomical form.

#### 4.1.2 Type II–bifurcated distal attachment

In this group, the FBT maintained its primary insertion at the lateral tuberosity of the fifth metatarsal, but also gave rise to an additional tendinous band with a distinct course and attachment site. This type was found in 30 lower limbs (29.4%) and subdivided as follows.

• Subtype IIa

The accessory band inserted into the dorsal surface of the base of the fifth metatarsal. At this point, it exhibited partial fusion with the tendon of fibularis tertius, suggesting a close ontogenetic relationship between the two structures. This configuration was found in 23 cases.

• Subtype IIb

The accessory band divided into two slips. The lateral slip inserted onto the dorsal surface of the fifth metatarsal base, while the medial, more prominent slip inserted into the midshaft of the same bone. This subtype was observed in five cases and may be of particular relevance in surgical exposure or trauma involving the shaft of the fifth metatarsal.

• Subtype IIc

This rarest variant involved an accessory band that, after bifurcation, formed a medial slip fusing with the fibularis tertius tendon. This fusion gave rise to an additional muscle belly interpreted as the fourth dorsal interosseous muscle (*musculus interosseus dorsalis IV*). This configuration was present in two cases.

These insertional variants are clinically significant due to their potential to mimic tendon pathology (e.g., longitudinal split tears) on MRI or United States, or to be misinterpreted intraoperatively. Moreover, the presence of connections with fibularis tertius underscores the importance of considering developmental muscle fusion when analyzing tendon morphology and planning lateral foot surgeries.

### 4.2 Co-occurring fibularis digiti quinti

The FDQ muscle was identified in 18 feet (17.7%) out of the 102 dissected lower limbs (Olewnik et al., 2020). In all cases, the presence of the FDQ tendon was strictly associated with the Type I configuration of the FBT. No instances of FDQ co-occurring with bifurcated (Type II) FBT insertions were recorded, suggesting a developmental link between these structures.

Three distinct types of FDQ were classified based on the location of the distal tendon insertion:

Type 1 – The FDQ inserted onto the base of the proximal phalanx of the fifth toe. This was the most common subtype and was present in eight cases. This configuration may contribute to subtle functional eversion of the fifth digit and should be considered in anatomical studies of lateral foot musculature.

Type 2 – The FDQ inserted into the extensor aponeurosis of the fifth toe. This subtype was found in five cases. The anatomical course in this variant aligns with the dorsolateral axis of the foot, and its presence may be misinterpreted on imaging as a duplication of extensor tendons.

Type 3 – The FDQ was fused with one of the tendons of the extensor hallucis longus (EHL) muscle. This uncommon configuration was observed in five feet. The fusion with EHL may reflect a developmental or phylogenetic relationship and represents an unusual deviation from the typical lateral foot anatomy.

All three variants are clinically relevant, particularly in surgical approaches to the lateral foot and during interpretation of radiological studies such as ultrasound or MRI. The FDQ may mimic pathological tendon structures, contribute to pseudomasses, or complicate soft-tissue dissections. Preoperative recognition of such variants can help prevent iatrogenic injury and improve surgical outcomes.

### 4.3 Historical classifications

The morphological diversity of the FBT has been noted for over a century. While current classification systems are based on detailed morphometric and imaging studies, earlier anatomical descriptions laid the groundwork for understanding the spectrum of insertional variability. Among the most influential early contributions are those by Macalister (1875), Bhargava et al. (1961), and Musiał (1963).

Macalister (1875) provided one of the first systematic surveys of muscular anomalies, including accessory tendons and fusion patterns in the lateral compartment of the leg. Although limited by the descriptive language of the time, his work documented numerous cases of accessory fibular insertions and connections with other foot musculature.

Bhargava et al. (1961), based on a study of 100 Indian cadavers, described the presence of split tendons and occasional dual insertions of the FBT. These observations closely resemble what would later be formalized as Type II configurations.

Musiał (1963) performed one of the earliest targeted studies of fibular muscle insertions in the Polish population, highlighting frequent deviations from the standard single insertion model. He reported examples of tendinous slips directed toward the cuboid, lateral cuneiform, or fascial structures now classified as Type II subtypes or atypical Type IV insertions.

To contextualize these foundational descriptions, the table below compares their major findings with the current classification system proposed by (Olewnik et al. (2020)) – Table 1.

**TABLE 1:** Comparison of historical and modern classifications of fibularis brevis tendon.

Source	Key observations	Corresponding Olewnik type
Macalister (1875)	Accessory fibular slips; fusion with fibularis longus; variable insertions	Type II, Type III
Bhargava et al. (1961)	Double insertions; split tendons	Type IIa, IIb
Musiał (1963)	Insertion to cuboid/lateral cuneiform/fascia; unusual branching patterns	Type IIc, Type IV
Olewnik et al. (2020)	Systematic typology: single vs. bifid insertion; fusion with FL; rare variants	Type I–IV

### 4.4 Morphological Development in Human Fetuses

A fetal anatomical investigation encompassing 84 lower limbs from 43 human fetuses (21 males, 22 females) revealed the consistent presence of the FBM and FBT (Olewnik et al., 2020). The study delineated three distinct tendon configurations during prenatal development, offering insights into the early organization and potential remodeling of the lateral ankle compartment (Olewnik et al., 2023).

Type I represented the predominant pattern, observed in 77% of specimens. It featured a single, direct insertion onto the lateral tuberosity at the base of the fifth metatarsal. This morphology mirrors the canonical adult form and likely reflects the default developmental trajectory in the absence of additional modulatory signals.

Type II, present in 19% of fetal limbs, exhibited a bifurcating tendinous pattern. While the main tendon consistently inserted in the typical location, secondary slips diverged in four anatomically distinct ways.

• Subtype A: A dorsal slip extended to the top surface of the fifth metatarsal base, often blending with fibers of the fibularis tertius tendon suggesting a shared developmental lineage or transient fusion.
• Subtype B: A secondary tendon anchored to the proximal shaft of the fifth metatarsal, introducing a more oblique force vector, potentially transient in function.
• Subtype C: The accessory band reached the fascia overlying the fourth interosseous space, implying a connection to dorsal structural stabilization.
• Subtype D: A unique tendinous fusion with the fibularis longus tendon was observed, indicating early topographic overlap and possible myotendinous reprogramming.

Type III, the rarest variant (4%), was characterized by a trifurcated distal arrangement. In this form, two accessory tendons accompanied the primary insertion: one attaching to the metatarsal shaft, and the other extending dorsally into fascial structures. This configuration, absent in adult morphotypes, may reflect an intermediate or regressing stage in fibular tendon morphogenesis.

These findings illustrate that the FBT undergoes dynamic refinement *in utero*, with several anatomical patterns likely subject to postnatal regression, fusion, or selective persistence. The existence of fetal-only variants such as Type III reinforces the notion of a developmental plasticity unique to this muscle group.

Clinically, such prenatal anatomical diversity bears significance in the context of congenital foot deformities, pediatric tendon dysplasia, or early-onset peroneal pathology. An appreciation of these morphotypes may aid in refining pediatric imaging protocols and guiding corrective interventions in infants. The observed fetal variants of the fibularis brevis tendon demonstrate a clear developmental continuum, with certain configurations showing direct correspondence to adult types, while others appear transient or prone to postnatal remodeling. A comparative summary of these relationships is presented in Table 2.

**TABLE 2:** Comparative summary of fetal and adult types of fibularis brevis tendon.

Fetal Type	Description	Adult correlate
Type I	Single insertion to lateral tuberosity of 5th metatarsal	Adult Type I
Type IIa	Accessory slip to dorsal base of 5th metatarsal; connects with fibularis tertius	Adult Type IIa
Type IIb	Accessory slip to proximal shaft of 5th metatarsal	Adult Type IIb
Type IIc	Accessory slip to fascia over 4th interosseous space	Adult Type IIc or IV
Type IId	Fusion with fibularis longus tendon	Adult Type III
Type III	Trifurcated: base, shaft, and fascia insertions	No direct adult analogue (transient)

## 5 Across species

The FBM demonstrates significant anatomical variability across vertebrate taxa, a reflection of ecological constraints, limb posture, and specific locomotor demands. The selection of species for the comparative analysis was guided by two principal criteria: phylogenetic diversity and limb posture classification. Specifically, we aimed to include representatives of both plantigrade and digitigrade locomotor types across major vertebrate clades to explore how evolutionary lineage and gait mechanics influence FBM morphology. Plantigrade species, such as humans, bears, and primates, maintain full-foot ground contact and generally require robust lateral stabilizers like the FBM. In contrast, digitigrade animals, including canines and felines, emphasize speed and efficiency by minimizing distal muscle mass, often resulting in reduced or fused FBM configurations. This framework provides a phylogenetically and functionally relevant basis for interpreting cross-species variability (Casteleyn et al., 2024; Holowka and Lieberman, 2018). In humans (*Homo sapiens*), FBM is a discrete and consistently present muscle that plays a crucial role in foot eversion and lateral ankle stabilization, especially in bipedal locomotion (Diogo and Abdala, 2007; Diogo and Abdala, 2010b; Diogo et al., 2009).

Among great apes such as *Pan troglodytes* and *Pongo pygmaeus*, FBM remains identifiable but frequently exhibits partial fusion with fibularis longus muscle. This architectural convergence likely represents an adaptation to arboreal behaviors, where strong yet integrated lateral control mechanisms are beneficial during brachiation or climbing (Diogo and Wood, 2012; Heilmann, 1926).

In carnivores like *Canis lupus familiaris*, FBM is well developed and independent from FL. It contributes to lateral stability during high-speed locomotion, particularly in agile cursorial species with digitigrade limb orientation (Diogo and Abdala, 2007; Diogo et al., 2010a; Diogo and Abdala, 2010b).

In contrast, the muscle is largely absent or fused in ungulates such as *Equus caballus*. These species exhibit a highly efficient sagittal-plane gait, which reduces the need for active eversion. Instead, passive elastic stabilization through fused fibular structures predominates (Diogo and Abdala, 2007; Diogo et al., 2010a; Diogo and Abdala, 2010b).

Rodents, including *Rattus norvegicus* and *Cavia porcellus*, show inconsistent FBM architecture. In many cases, FBM is rudimentary or entirely absent, with FL compensating as the main evertor and lateral stabilizer (Diogo and Abdala, 2007; Diogo et al., 2010a; Diogo and Abdala, 2010b).

Among marsupials, FBM morphology varies significantly. In terrestrial hoppers like *Macropus rufus* (red kangaroo), FBM is often reduced or redirected, while in arboreal species such as *Phalanger* or *Trichosurus*, a more robust FBM is present, supporting precise control of foot position on variable substrates (Diogo and Abdala, 2007; Diogo et al., 2010a; Diogo and Abdala, 2010b).

In avian species such as *Gallus* (domestic chicken), FBM is entirely absent, consistent with the evolution of a digitigrade limb used primarily for perching and straight-line motion. Lateral stability is instead maintained by tendinous expansions of FL and the lateral crural fascia (Diogo and Abdala, 2007; Diogo et al., 2010a; Diogo and Abdala, 2010b).

Reptiles, including *Varanus* and *Iguana*, do not possess a true FBM muscle. However, serially homologous muscle groups originating from the lateral dorsal myotomes are present, forming the phylogenetic groundwork from which FBM may have evolved in tetrapods (Diogo and Abdala, 2007; Diogo et al., 2010a; Diogo and Abdala, 2010b).

**FIGURE 1:**
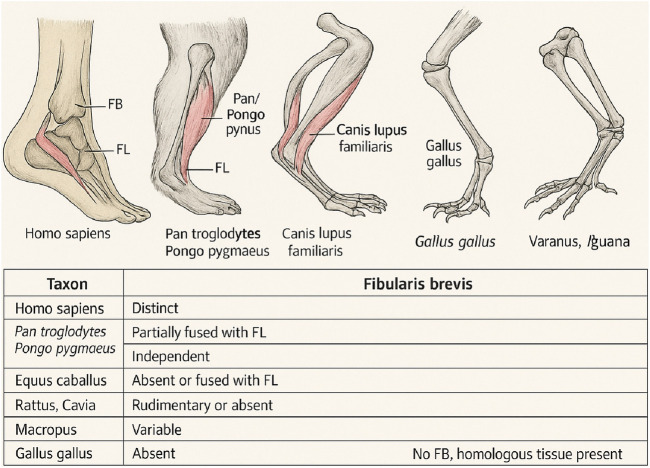
Comparative anatomical status of the fibularis brevis muscle across selected vertebrate taxa.

These findings indicate that the presence, fusion, or absence of FBM strongly correlates with mechanical specialization and phylogenetic heritage. Arboreal species tend to retain a distinct and hypertrophied FBM, cursorial mammals show simplification or fusion, and taxa with reduced lateral foot demands tend to lose this muscle altogether. A comparative overview of fibularis brevis presence and morphology across selected vertebrate groups is presented in Figure 1.

A detailed summary of fibularis brevis across species is presented in Table 3.

**TABLE 3:** Comparative presence and morphological status of fibularis brevis across taxa.

Species/Taxon	FB presence	Functional interpretation
Homo sapiens	Distinct and consistent	Eversion and ankle stabilization in bipedal gait
Pan troglodytes	Partially fused with FL	Adaptation for climbing; integrated control
Pongo pygmaeus	Often fused with FL	Climbing adaptation; reduced independence
Canis lupus familiaris	Well-developed, independent	Lateral stability during high-speed movement
Equus caballus	Absent or fused	Sagittal efficiency; fusion with FL structures
Rattus/Cavia	Rudimentary or absent	FL compensates for lateral control
Macropus	Reduced or redirected	Simplified eversion; strong propulsion
Phalanger/Trichosurus	Robust, hypertrophied	Precise substrate grip in arboreal gait
Gallus gallus	Absent	Passive stability via tendinous structures
Varanus/Iguana	No true FB; homologous myosepta	Primitive patterning; evolutionary precursors

### 5.1 Functional morphology

The architecture of the FBM demonstrates strong functional alignment with the specific locomotor demands imposed by an organism’s ecological niche. Across vertebrate taxa, variations in muscle size, shape, insertion pattern, and degree of independence from fibularis longus reflect adaptations that optimize mechanical efficiency, stability, or precision.

In arboreal species, such as *Pan troglodytes* and *Phalanger* spp., FBM tends to be hypertrophied and well-differentiated, often maintaining an independent myotendinous trajectory. This configuration supports powerful foot eversion and active modulation of grasp during branch manipulation. The hypertrophy correlates with dynamic stabilization demands in climbing and suspension behaviors (Diogo et al., 2010a; Heilmann, 1926).

In cursorial mammals, including *Canis lupus familiaris* and *Equus caballus*, the FBM shows either increased fusion with FL or complete anatomical reduction. In canines, FBM remains functional and distinct, assisting with lateral foot stabilization during rapid directional changes. In ungulates, where limbs are optimized for straight-line, high-speed movement, FBM is commonly fused or absent, replaced by robust tendinous extensions of FL. This streamlining promotes energy-efficient elastic recoil and reduces muscular redundancy (Abdala and Diogo, 2010; Diogo et al., 2010a; Diogo and Abdala, 2010b).

Marsupials and climbers, such as *Macropus rufus* and *Trichosurus vulpecula*, exhibit variable FBM morphology, tuned to their respective locomotor strategies. Terrestrial hoppers show a simplified FBM, likely serving a limited proprioceptive or supportive role, whereas arboreal marsupials preserve a more distinct FBN for lateral control and postural adjustment on irregular substrates (Abdala and Diogo, 2010; Diogo et al., 2010a; Diogo and Abdala, 2010b).

Functionally, FBM’s anatomical variability affects.

• Tendon trajectory: direct vs. bifurcated insertions influence torque vectors on the fifth metatarsal.
• Muscle belly orientation: deeper or superficial placement affects mechanical advantage in eversion.
• Fascial compartmentalization: independence from fibularis longus facilitates finer control, whereas shared sheaths increase efficiency but reduce flexibility.

These patterns underscore FBM’s evolutionary role as a modular structure selectively retained, remodeled, or minimized depending on biomechanical demands. Its form serves as both a reflection of locomotor ecology and a potential marker of developmental constraints. In addition to morphological diversity across taxa, the insertional pattern of the FBT itself particularly bifurcated configurations may have meaningful biomechanical implications. The FBM is responsible for approximately 60%–65% of the eversion force generated at the ankle. A bifurcated insertion pattern, involving multiple attachment sites, can provide a broader distribution of tensile force across the lateral foot, thereby enhancing subtalar joint stability and resistance to inversion stress. Conversely, a single-point (Type I) insertion concentrates mechanical loading at the base of the fifth metatarsal, which may increase the risk of focal degeneration or avulsion injuries. These biomechanical considerations underscore the potential clinical significance of anatomical variants, particularly in populations engaged in high-impact or lateral-movement sports (Olewnik et al., 2020). Subtle distinctions in FBM structure are also evident when comparing limb postures across taxa. In plantigrade species such as humans, bears, and primates the FBM tends to be more robust and muscular, reflecting its crucial role in stabilizing the entire foot during full-sole contact with the ground. In contrast, digitigrade animals like dogs and cats often exhibit a more streamlined, tendon-dominated FBM configuration. Their foot posture, which emphasizes toe-based contact and high-speed locomotion, favors mechanical efficiency over lateral fine-control. These morphological nuances align with broader locomotor adaptations and underscore the evolutionary plasticity of FBM in response to functional demands.

### 5.2 Evolutionary and clinical implications

Understanding the evolutionary origins and morphofunctional variability of the FBM provides important insights for both developmental biology and clinical anatomy. Variant morphologies observed in humans may reflect retained ancestral traits or deviations arising during embryonic patterning.

#### 5.2.1 Human FB fusion types as potential atavistic configurations

Fused forms of FBM with fibularis longus, or bifid insertions, may represent evolutionary echoes of more primitive muscular arrangements found in other mammals. Comparative studies have shown that fusion of FBM with fibularis longus is common among species where lateral foot mobility is reduced or simplified. Therefore, fusion variants in humans might not be merely anatomical curiosities, but rather vestiges of older muscular templates conserved through phylogeny (Abdala and Diogo, 2010; Diogo et al., 2010a; Diogo and Abdala, 2010b; Diogo and Wood, 2012).

#### 5.2.2 Developmental anomalies as reflections of ancestral states

Variants such as bifurcated tendons, accessory fascicles, or unusual insertions seen in fetuses (Olewnik et al., 2023) might reflect incomplete remodeling during postnatal development. These anomalies often correspond to muscular arrangements documented in non-human mammals or earlier developmental stages, reinforcing the link between ontogeny and phylogeny. Such variants may persist into adulthood and manifest clinically.

#### 5.2.3 Misdiagnosis risk due to variant FB morphology during MRI interpretation

The clinical implications of FBM variants are significant, particularly in radiological imaging. Bifid tendons or accessory slips can mimic longitudinal tendon tears or soft tissue masses, leading to misinterpretation on MRI. Awareness of such variants is essential for avoiding diagnostic errors, especially in patients presenting with lateral ankle pain or instability (Sookur et al., 2008; Zielinska et al., 2024).

#### 5.2.4 Surgical planning

Anatomical variability of FBM may also complicate lateral surgical approaches to the ankle. Variants with multiple insertions, accessory heads, or close proximity to neurovascular structures increase the risk of iatrogenic damage. In tendon harvesting procedures or during lateral ligament reconstruction, unrecognized FBT variants may lead to suboptimal graft preparation or unintended compromise of surrounding tissues (Olewnik et al., 2020; Ruzik et al., 2022).

#### 5.2.5 Role of evolutionary anatomy in refining surgical landmarks

Integrating evolutionary and comparative anatomy into clinical practice offers a refined understanding of muscular landmarks. Knowledge of which FBM morphologies are likely to occur based on phylogenetic context can help surgeons anticipate unexpected patterns during dissection or imaging. This enhances surgical safety, improves diagnostic specificity, and fosters a deeper anatomical literacy among clinicians.

## 6 Imaging, radiological pitfalls, and protocol suggestions

### 6.1 Normal and variant FB appearance in MRI and ultrasound

The FBT typically appears as a low-signal-intensity structure on T1- and T2-weighted MRI sequences, located anteromedial to the fibularis longus in the lateral retromalleolar groove. It maintains a uniform thickness and course, inserting on the base of the fifth metatarsal. In United States, FBT demonstrates a fibrillar echotexture and lies within a shared peroneal sheath, becoming hypoechoic in longitudinal tears or tenosynovitis (Buckens et al., 2017; Sookur et al., 2008). Variant forms, such as bifurcated tendons or anomalous insertions, may alter the tendon trajectory or thickness, often appearing as accessory bundles or subtle duplications within the sheath (Zielinska et al., 2024).

### 6.2 Common diagnostic errors: bifid FBT, FDQ, accessory muscles

Several FBM variants may mimic pathologic conditions on imaging. A bifid FBT, especially when the accessory slip is hypoplastic or curved, may simulate a longitudinal split tear on both MRI and United States, leading to unnecessary interventions. The FDQ tendon, when present, can appear as a small independent structure extending towards the fifth toe, and may be misinterpreted as a retracted tear or neuroma. Similarly, accessory fibularis muscles such as the peroneus quartus or fibularis accessorius may be mistaken for masses, particularly in the retromalleolar or calcaneal regions (Sookur et al., 2008; Yammine, 2015). Awareness of these variations is essential in patients presenting with lateral ankle pain, especially among athletes and individuals with overuse injuries.

### 6.3 Imaging guidelines: dynamic United States, slice orientation, signal characteristics

To accurately differentiate variants from pathology, optimized imaging protocols are necessary. Dynamic United States should be used to assess for intrasheath subluxation, retinacular impingement, or split tendon motion. The ankle should be scanned in both neutral and dorsiflexed positions. For MRI, axial and oblique axial slices are preferred at the level of the distal fibula and cuboid, with high-resolution proton-density (PD) and STIR sequences for tendon evaluation. Key parameters include tendon thickness asymmetry >2 mm, signal heterogeneity, and anomalous insertion paths (Bencardino and Rosenberg, 2001; Mateen et al., 2022). For optimal visualization of FBT variants, we recommend a dedicated MRI protocol using proton-density (PD) or T2-weighted sequences with fat suppression. Axial-oblique slices aligned with the peroneal tendons particularly behind the lateral malleolus are ideal for detecting bifurcated or accessory slips. Coronal-oblique planes oriented parallel to the fifth metatarsal further aid in assessing insertional morphology. Ultrasound, when performed dynamically during active foot eversion, serves as a valuable adjunct for confirming variant tendons in real time and for assessing their relationship to surrounding structures (Wang et al., 2005).

### 6.4 Recommendations for radiologists and sports medicine specialists

Radiologists should consider anatomical variability in differential diagnoses of lateral ankle lesions, particularly when evaluating for peroneal tendon tears, tenosynovitis, or suspected soft tissue masses. When an unexpected bifid structure is noted, symmetry and continuity of both slips should be confirmed before diagnosing a tear. Sports medicine specialists and orthopedic surgeons should correlate imaging findings with clinical symptoms and physical examination, especially when considering surgical intervention. Preoperative imaging should aim to define not only pathology but also variant tendon anatomy to guide incision placement and graft viability (Heckman et al., 2008; Olewnik et al., 2020).

## 7 Clinical applications and surgical risk mapping

### 7.1 FBT in peroneal tendon pathologies: tenosynovitis, tears, instability

The FBT is commonly implicated in lateral ankle pathologies, including tenosynovitis, longitudinal tendon tears, and chronic ankle instability. FBT injuries often co-occur with peroneal tendon sheath disorders and are particularly prevalent among athletes. According to Mercer et al. (Mercer et al., 2021), surgical intervention for peroneal tendon tears, including FBT, led to favorable functional outcomes but was associated with a complication rate nearing 38.7%. Low-lying muscle bellies and bifurcated tendons may predispose to chronic friction and tendon degeneration (Sookur et al., 2008). These variants have also been shown to increase the risk of tenosynovitis and peroneal tendon subluxation (Koutsogiannis et al., 2022).

### 7.2 Anatomical variants: influence on surgical exposure, graft harvesting, retraction risk

Anatomical variation of the FBT can complicate both diagnosis and surgical planning. For instance, bifid FBT may be mistaken for a split tear, and fusion with fibularis longus tendon can obscure clear visualization during tendon transfers or tenodesis procedures. Moreover, harvesting FBT or fibularis longus tendon for autografts in lateral ankle ligament reconstruction may endanger peroneal vincula, which are critical for vascular supply to the tendon (van Dijk et al., 2016). Ruzik et al. (Ruzik et al., 2022) reported that an unusual FBT insertion pattern may require modified dissection planes to prevent neurovascular injury, particularly to the sural nerve and small saphenous vein.

### 7.3 Risk stratification and identification: intraoperative strategies

Preoperative imaging with ultrasound or MRI can help identify FBT variants, especially bifid insertions or anomalous accessory fascicles. During surgery, dynamic assessment of tendon movement and visual confirmation of insertion sites is critical. When variants are suspected, careful blunt dissection and extension of the surgical window may reduce the risk of iatrogenic injury. Tendon tension testing under anesthesia can help confirm integrity and differentiate between FBT and adjacent slips (Bianchi et al., 2010; Bianchi et al., 2007; Heckman et al., 2008).

### 7.4 Preoperative planning and proposed “surgical safety map”

A tailored approach is necessary for surgical procedures involving FBT, particularly in reconstructive contexts. We propose a safety-oriented preoperative checklist: (1) assess FBT morphology via imaging; (2) localize adjacent neurovascular structures (especially the sural nerve); (3) confirm absence or presence of FDQ or fibularis quartus muscles. Imaging should guide incision planning, and knowledge of variant configurations should inform intraoperative navigation. Such an approach reduces the likelihood of incomplete graft harvesting or inadvertent soft tissue damage.

## 8 Terminological and taxonomic refinement

### 8.1 Unification of nomenclature: FBM, FBT, FDQ, fibularis quartus

The terminology surrounding the lateral compartment of the leg particularly in reference to FBM, its tendon, the FDQ, and accessory muscles such as the fibularis quartus remains inconsistent across anatomical atlases, radiological reports, and surgical literature. While classical texts such as those by Macalister (Macalister, 1875) and Anson (Anson and McVay, 1971) provided descriptive terms, they often lacked uniformity in defining morphological variants versus distinct muscular entities. Modern anatomical works (e.g., Bergman et al. (Bergman et al., 1988)) have attempted to consolidate these terms, but considerable confusion remains, particularly in distinguishing FDQ from fibularis quartus or fibularis accessorius.

### 8.2 Rationale: comparative anatomy, embryology, radiological practice

The need for standardized nomenclature arises from both evolutionary and clinical perspectives. From an evolutionary viewpoint, Diogo and Abdala (Diogo et al., 2010a; Diogo and Abdala, 2010b) have emphasized the modular origin of the FBM and its relation to digital extensor systems in primitive tetrapods, which supports the view that FDQ and fibularis quartus are derived subdivisions rather than separate muscles. Embryologically, these structures share a common origin from the dorsal muscle mass of the limb bud and differentiate under the influence of Pax3, Myf5, and MyoD (Albay and Candan, 2017; Bardeen, 1906; Bardeen and Lewis, 1901). Clinically, imaging artifacts due to anatomical variability such as bifid tendons or partial fusions are often misinterpreted in MRI and United States, underscoring the need for precise, consistent descriptors (Sookur et al., 2008; Zielinska et al., 2024).

### 8.3 Proposal for standardized anatomical terminology

We propose the following standardized terminology.

• Fibularis brevis muscle should refer exclusively to the muscle originating from the distal lateral fibula, inserting on the fifth metatarsal, regardless of its internal subdivisions.
• Fibularis brevis tendon should describe the distal tendinous portion, including both single and bifurcated insertions as defined in Type I–IV classification (Olewnik et al., 2020).
• Fibularis digiti quinti should be reserved for a distinct accessory tendon inserting on the fifth toe, regardless of its origin.
• Fibularis quartus should refer only to independent muscular bellies that originate from FBM or fibularis longus and insert onto the retrotrochlear eminence or calcaneus, clearly separable from FDQ and from fascicular variants of FBT.

This classification aims to improve clarity across disciplines, facilitate radiological reporting, and support more precise anatomical education. Adoption of uniform descriptors in anatomical atlases and surgical protocols will reduce misdiagnosis and improve patient outcomes in cases involving lateral ankle pathology.

## 9 Final synthesis: unified classification framework

### 9.1 Morphological summary: types I–IV + FDQ

The FBT exhibits a range of morphological patterns, classified into four main types based on distal insertion, as described by Olewnik et al. (Olewnik et al., 2020) and further elaborated in fetal and adult anatomical studies.

• Type I: Single distal attachment to the tuberosity of the fifth metatarsal.
• Type II: Bifid insertion with subtypes A–E, depending on the accessory band attachment site (e.g., dorsal surface, shaft, fascia, FL fusion).
• Type III: Fusion with fibularis longus.
• Type IV: Atypical insertion or trifurcation pattern.

Additionally, the FDQ tendon occurs in approximately 18% of limbs and is closely associated with Type I FBT, with its own subclassification based on insertion site (e.g., proximal phalanx, extensor aponeurosis, extensor hallucis longus tendon fusion).

### 9.2 Developmental and phylogenetic context

The classification integrates ontogenetic insights from fetal studies, where early developmental patterns often regress or remodel into adult configurations (Olewnik et al., 2023). Type II variants in adults likely reflect incomplete postnatal remodeling or persistence of accessory bands originally present in fetal Type II or III patterns. From a phylogenetic standpoint, FBT variability mirrors adaptations across species: hypertrophy in arboreal primates, fusion or reduction in ungulates and cursorial mammals, and vestigial absence in certain rodent taxa (Diogo, 2005; Diogo, 2007; Diogo, 2010; Diogo et al., 2010a; Heilmann, 1926).

### 9.3 Clinical and imaging correlates

Anatomical variants of FBT and FDQ are clinically significant. Bifid tendons may be mistaken for partial tears or soft-tissue masses on MRI or United States (Bianchi et al., 2010; Bianchi et al., 2007; Sookur et al., 2008). FDQ can mimic anomalous muscle bellies or accessory tendons. Awareness of these patterns is essential in preoperative imaging and intraoperative navigation, particularly in reconstructive procedures or tendon transfers involving the peroneal group. Variant configurations may also increase the risk of tenosynovitis or nerve entrapment due to altered biomechanics or fascial crowding.

### 9.4 Tabular integration of all data sources

To consolidate morphological, developmental, evolutionary, imaging, and clinical data, we propose a unified table (Table 4) summarizing each FBT and FDQ type. This integrative tool provides.

• Anatomical description and frequency
• Fetal analogs and phylogenetic parallels
• Imaging appearance and diagnostic pitfalls
• Surgical implications and risk mapping

**TABLE 4:** Unified classification of fibularis brevis tendon (FBT) and fibularis digiti quinti (FDQ).

Type	Anatomical description	Fetal analogy	Imaging considerations	Clinical implications
Type I	Single insertion on base of 5th metatarsal	Fetal Type I	Normal MRI/USG pattern	Standard anatomy for reconstruction
Type IIa	Accessory band to dorsal surface of base	Fetal Type IIa	May mimic partial tear on MRI	Risk of surgical misidentification
Type IIb	Accessory band to shaft of 5th metatarsal	Fetal Type IIb	May appear as split tendon	May predispose to tenosynovitis
Type IIc	Accessory to interosseous fascia	Fetal Type IIc	Difficult to visualize; risk of misinterpretation	Fascial entrapment risk
Type IId	Accessory fused with FL tendon	Fetal Type IId	Can be mistaken for accessory tendon	Confounding during graft harvest
Type IIe	Rare or complex insertions (e.g., trifurcated)	Fetal Type III	Variant signal; requires expert interpretation	Requires customized surgical approach
Type III	Fusion with fibularis longus	No distinct fetal correlate	May obscure FL/FB boundary	Changes plane of dissection
Type IV	Atypical, including trifurcation patterns	Fetal Type IV	Unusual signal morphology	Surgical anomaly; rare need for adaptation
FDQ Type 1	Insertion to base of proximal phalanx of 5th toe	Associated with Fetal Type I	Mimics accessory tendon or soft tissue mass	May cause tendon crowding symptoms
FDQ Type 2	Insertion to extensor aponeurosis of 5th toe	Associated with Fetal Type I	Subtle; often not visible on US	Low surgical impact but important diagnostically
FDQ Type 3	Fusion with extensor hallucis longus tendon	Associated with Fetal Type I	Potential confusion with extensor tendons	Rare; potential misdiagnosis as pathology

Such a classification framework can enhance anatomical education, improve diagnostic accuracy, and guide surgeons in pre- and intraoperative decision-making–Table 4.

## 10 Future directions

The FBM should no longer be regarded as a simple, monomorphic structure but as part of a morpho-evolutionary continuum, reflecting both developmental plasticity and phylogenetic modulation. Future anatomical and clinical research should aim to clarify the extent, significance, and application of these variations.

Recommended areas of study include.

• High-resolution MRI and ultrasound prevalence studies focused on variant forms of FBT and FDQ in different populations, including pediatric and athletic cohorts.
• Electromyographic (EMG) analysis across gait phases, to investigate the dynamic contribution of FBT in various anatomical configurations.
• AI-based segmentation and anatomical modeling for FBT and its variants to improve diagnostic accuracy and surgical planning in musculoskeletal imaging (including deep learning protocols).

Standardization of imaging protocols, cadaveric dissection methodology, and classification criteria will be key for cross-study comparability and for translating anatomical knowledge into clinical benefit.

## 11 Limitations

This review, while comprehensive, is subject to several limitations.

• Heterogeneity of available data: Existing studies vary widely in dissection protocols, imaging modalities, and inclusion criteria, making direct comparison challenging.
• Lack of large-scale prevalence data: Most anatomical classifications of FBT and FDQ are based on small sample sizes; larger multicenter studies are needed.
• Limited functional validation: Although variant forms have been described morphologically, few studies have linked these to functional gait analyses or biomechanical testing.
• Comparative data gaps: While evolutionary perspectives are included, some taxa (e.g., marsupials, rodents, birds) remain underrepresented in the anatomical literature.

## 12 Conclusion

The fibularis brevis muscle should be redefined as a variable, developmentally regulated, and evolutionarily significant component of the lateral leg compartment. Its tendon demonstrates consistent yet diverse patterns of insertion, often accompanied by accessory structures such as the fibularis digiti quinti.

Anatomical awareness of these variants is essential for avoiding misinterpretation in imaging, improving surgical accuracy, and expanding our understanding of locomotor evolution. Through the integration of embryology, comparative anatomy, radiology, and clinical science, FBT can serve as a model for morpho-functional research in musculoskeletal medicine.
